# A Sample and Sensitive HPLC-MS/MS Method for Simultaneous Determination of Ziyuglycoside I and Its Metabolite Ziyuglycoside II in Rat Pharmacokinetics

**DOI:** 10.3390/molecules23030543

**Published:** 2018-02-28

**Authors:** Zhi-Feng Li, Meng-Ying Zhou, Ting Tan, Chen-Cong Zhong, Qi Wang, Ling-Ling Pan, Ying-Ying Luo, Shi-Lin Yang, Yu-Lin Feng, Hui Ouyang

**Affiliations:** 1 National Pharmaceutical Engineering Center for Solid Preparation in Chinese Herb Medicine, Jiangxi University of Traditional Chinese Medicine, Nanchang 330004, China; kjxykfzl2014@126.com (Z.-F.L.); zmy1005483476@163.com (M.-Y.Z.); snow145@163.com (T.T.); m18720062807@163.com (C.-C.Z.); 18300687731@163.com (Q.W.); fdh528508@163.com (L.-L.P.); yangshilin@suda.edu.cn (S.-L.Y.); 2 State Key Laboratory of Innovative Drug and Efficient Energy-Saving Pharmaceutical Equipment, Jiangxi University of Traditional Chinese Medicine, Nanchang 330006, China; fdhzhaolanjun@163.com

**Keywords:** ziyuglycoside I (ZGS1), ziyuglycoside II (ZGS2), LC-MS/MS, pharmacokinetics, tissue distribution, excretion

## Abstract

Ziyuglycoside I (ZGS1) is a promising drug candidate for the treatment of leucopenia. Currently, information on ZGS1 and its in vivo metabolite ziyuglycoside II (ZGS2) is limited. The objective of this study was to investigate the pharmacokinetics, tissue distribution, and excretion of ziyuglycoside I (ZGS1) and its metabolite ziyuglycoside II (ZGS2) in rats. In our study, a simple and sensitive high-performance liquid chromatography-mass spectrometry (HPLC-MS/MS) method was established for simultaneous determination of ZGS1 and its metabolite for Sprague-Dawley rat pharmacokinetics studies. The method was validated following internationally-approved guidelines. The results presented in this study indicated that subcutaneous administration of ZGS1 prolonged its extension time and increased the area under the curve (AUC_0–t_) of ZGS2 during 0 to t minutes. In summary, in this study, the pharmacokinetic characteristics of ZGS1 and its metabolite ZGS2 were defined and its tissue distribution, and excretion in rats were described. Our finding may be beneficial for leucopenia drug that focus on ZGS1.

## 1. Introduction

In recent years, cancer incidence rates have risen worldwide. Currently, the health problems caused by cancer treatment are receiving increased attention. Chemotherapy is an important method of therapy in the treatment of malignancies. Different chemotherapeutic agents have different levels of myelosuppression, which may limit the dose that can be used, and can easily result in interruption of chemotherapy. This serious side effect can significantly affect the treatment of malignancies and decrease the quality of a patient’s life. At present, granulocyte colony-stimulating factor is used as adjuvant therapy for the reduction of neutropenia. However, granulocyte colony-stimulating factor also has side effects, including bone pain, and flushing [1,2,3,4,5].

In the clinic, extracts from traditional Chinese medicine have received increased attention for their favorable pharmacodynamics effects and low toxicity. In China, burnet root leukopoietic tablets (BRLT) have been widely used due to their beneficial effects on leucopenia caused by tumor-targeting chemotherapy. In previous studies, it was demonstrated that in patients who were given BRLT, the incidence of chemotherapy-induced myelosuppression, was significantly reduced [6,7]. BRLT are prepared from *Sanguisorba officinalis* L., a traditional Chinese herb that has been used in China for over 3000 years. BRLT have shown beneficial effects in a variety of disease, including infection, inflammatory, cancer, allergic, and diseases of the central nervous system [8]. Previous studies have demonstrated that *Sanguisorba* contains bioactive constituents of saponins and tannins [9]. Several clinical reports have demonstrated a pre-protective role of *S. officinalis* on leucopenia [10,11]. Moreover, it has been shown that saponins extracted from this plant reduce myelosuppression and 60 Co-γ-irradiation in mice [12]. ZGS1 is the main saponin exacted from the famous traditional Chinese medicine [13], and has the ability to inhibit apoptosis of TF-1 cells, reduce mouse cytokine production, and promote the survival of focal adhesion kinase (FAK) mouse bone marrow nuclear cells in vitro [14,15,16,17]. In our previous study, we showed that ZGS2 is a metabolite of ZGS1 that protects against the appearance of marrow repression during tumor chemotherapy. Therefore, ZGS1 has been chosen as a novel leucopenia drug and needs further research. Currently, limited information is available on ZGS1 and its metabolite ZGS2 in vivo. Therefore, it is of great importance to investigate these compounds and their pharmacokinetics. 

The pharmacokinetic profile of the prototype and their metabolites is a critical estimation of drug candidates. A novel quantitative method for ZGS1 and ZGS2 was established using an Agilent 6460 instrument in this study. This is the first profile study describing the absorption, distribution, and excretion of ZGS1 and ZGS2, and the first time that the pharmacokinetic profile of ZGS1 and its metabolite ZGS2 were investigated. This study provides novel insights for future clinical practice of ZGS1 and ZGS2, which may be promising for the development of ZGS1-based therapeutics in treating leucopenia.

## 2. Results and Discussion

### 2.1. Method Development

**Figure 1:**
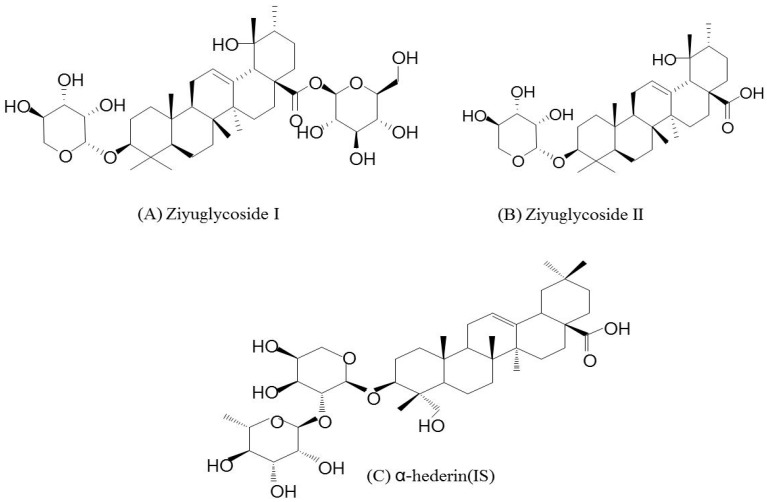
Chemical structures of (**A**) ZGS1; (**B**) ZGS2; and (**C**) α-hederin (internal standard).

To obtain better and interference-free extracts, protein precipitation was used and several solvents were studied. During the precipitation procedure, the use of methanol, acetonitrile, and methanol containing 0.1% FA were investigated. Among the solvents, the addition of 0.1% FA to methanol significantly promoted the recovery of analytes in biological samples. Moreover, the use of the XTERRA MS column gave a sharp peak for ZGS1, ZGS2, and the IS. The chemical structures of all compounds are presented in Figure 1. In the chromatographic separation of analytes, acetonitrile gave a better peak shape and had a lower background when compared to methanol. In comparison with acetic acid and ammonium acetate, the addition of 0.1% FA in methanol significantly enhanced the shapes of the peaks. Therefore, acetonitrile and water (with 0.2% FA) were adopted as the mobile phases. In addition, a gradient elution was chosen to reduce interference of the endogenous substance.

**Figure 2:**
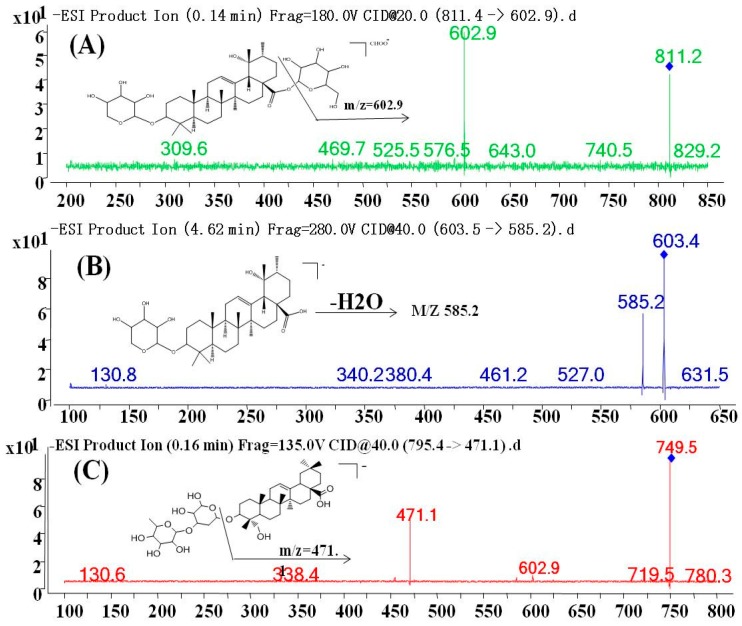
Fragment ion spectra of (**A**) ZGS1; (**B**) ZGS2; and (**C**) α-hederin (internal standard).

MS parameters were optimized by injecting standard solutions into the MS. For the optimization of ESI conditions for ZGS1, ZGS2, and IS, quadrupole full scans were performed in both positive (+) and negative (−) ion detection modes. Due to the higher sensitivity and a more stable response, the negative mode was more suitable for ZGS1, ZGS2, and the IS. To ascertain precursor ions, ZGS1, ZGS2, and the IS were initially characterized by a tandem mass spectrometry Q1 (full mass spectra) scan. Amproduct scan was used for the selection of suitable product ions, and was used in the MRM mode. Full mass spectra of ZGS1, ZGS2, and the IS, as well as their MS/MS spectra are shown in Figure 2. The MRM transitions of ZGS1, ZGS2, and the IS were *m*/*z* 811.4→602.9, *m*/*z* 603.5→585.3, and *m*/*z* 749.3→471.1, respectively. The fragment and CE were optimized to improve the sensitivity of the detection of the precursor and product ions (Table 1). Other mass spectrometry-related parameters, such as ion spray voltage (ISV), capillary temperature (CT), nebulizer gas (GS1), auxiliary gas (GS2), curtain gas (CUR), and collision gas (CAD) were also optimized to achieve a better response.

### 2.2. Internal Standard

After evaluating the retention time, peak shape, response, and MF, α-hederin was chosen as the IS, which met the requirements of the analysis.

### 2.3. Method Validation

#### 2.3.1. Calibration Curve, LLOQ, Carryover, and Specificity

**Figure 3:**
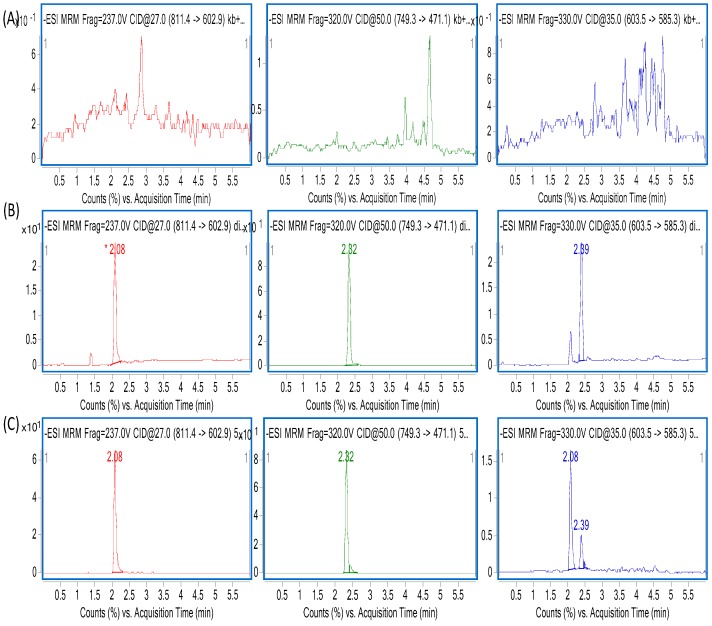
Representative MRM chromatograms of ZGS1, α-hederin (internal standard [IS]) and ZGS2 in (**A**) blank plasma sample; (**B**) blank plasma sample spiked with LLQC of ZGS1 (10 ng/mL) and ZGS2 (10 ng/mL), and IS (800 ng/mL); (**C**) rat plasma sample obtained 1h after subcutaneous injection of ZGS1 (10 mg/kg).

Over a range of 5–2000 ng/mL, both ZGS1 and ZGS2 showed a good linearity. Calibration curves were evaluated using a quadratic equation with a weighting factor: WF = 1/*x*^2^. The correlation coefficient of all calibration curves was >0.99 and the concentration of the samples used for preparation of the calibration curve was within 85–115% of the expected value (Table 2 and Table 3). Representative specificity MS chromatograms for blank and LLOQ plasma samples are shown in Figure 3. When blank plasma sample extracts were injected immediately after injection of the ULOQ sample, no significant carryover was observed. Moreover, no significant interference from endogenous substances was observed, indicating that the specificity of the method was sufficient.

#### 2.3.2. Precision and Accuracy

As shown in Table 4, the results obtained from the method validation met the FDA criteria [18]. The Intra-day and Inter-day accuracy of all analytes was <6%. In addition, the precision of all analytes ranged from 0.81% to 4.48%, indicating that the method had a good precision and accuracy.

#### 2.3.3. Recovery and Matrix Effect

Table 5 summarizes the extraction recovery (ER) and ME of ZGS1 and ZGS2 in rat plasma. The ER of all analytes ranged from 96% to 102%, indicating that the recoveries met the requirements. ME were evaluated by using MF and IS-normalized MF. Among the three evaluated QC levels, the IS-normalized MF ranged from 100% to 107%. These findings indicated that there were no problems in the MF of analytical methods of ZGS1 and ZGS2.

#### 2.3.4. Stability

As shown in Table 6, the analytes were stable after incubating at room temperature for at least 24 h (short-term stability), three freeze-thaw cycles, and after storage for 30 days at −80 °C (long-term stability). Thus, these results demonstrated that ZGS1 and ZGS2 are stable at different storage conditions.

#### 2.3.5. Dilution Integrity

The accuracy and precision of the dilution reliability of ZGS1 in plasma were 6.58% and 3.48%, respectively. This indicated that samples have a higher concentration than the ULOQ could predict and quantitatively in analysis after dilution with a blank matrix.

### 2.4. Pharmacokinetic Study

**Figure 4:**
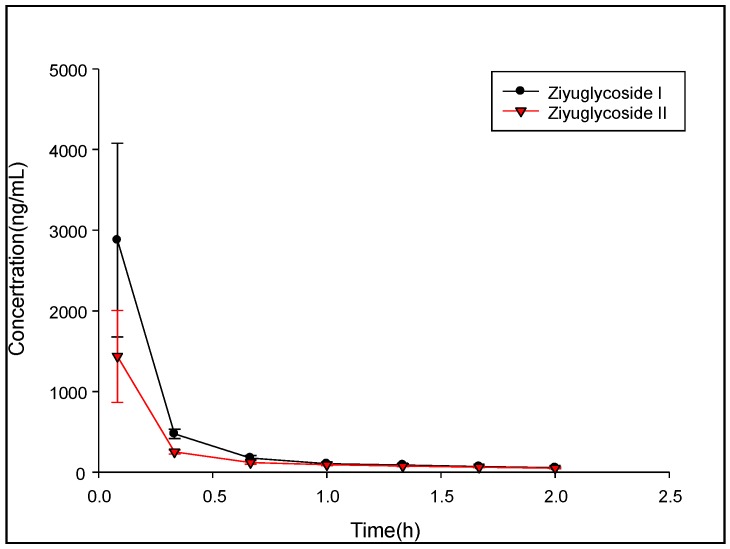
Mean plasma concentration-time profiles of ZGS1 and ZGS2 after tail vein administration of ZGS1 (10 mg/kg) (*n* = 5).

**Figure 5:**
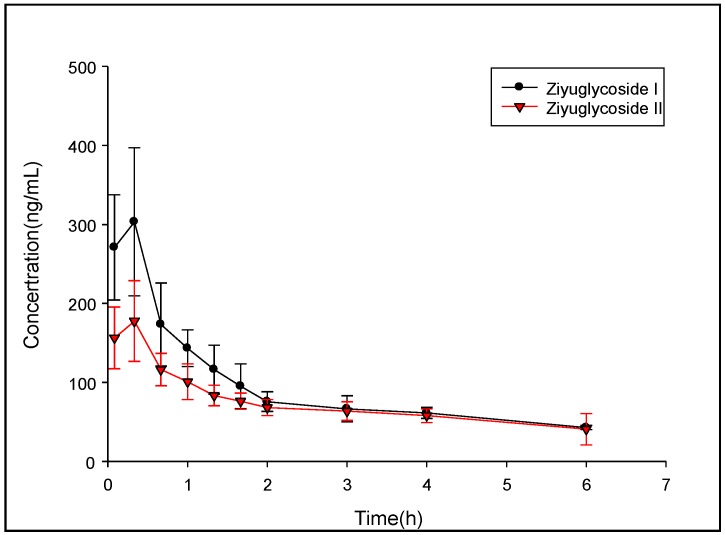
Mean plasma concentration-time profiles of ZGS1 and ZGS2 after subcutaneous injection administration of ZGS1 (10 mg/kg) (*n* = 5).

The pharmacokinetic (PK) study of ZGS1 and ZGS2 was performed in ten male SD rats weighing 150–200 g. The PK parameters were evaluated by a non-compartmental model (DAS 3.0 software). PK parameters represented the average values of 5 rats (Figure 4 and Figure 5). The half-life (t_1/2_) of tail vein-administrated ZGS1 was 1.338 ± 0.744 h and 1.027 ± 0.057 h for the active metabolite ZGS2. Moreover, t_1/2_ of subcutaneously administered ZGS1 was 6.115 ± 1.92 and 7.935 ± 3.264 h for ZGS2 (Table 7 and Table 8). The relative bioavailability of ZGS1 when administered subcutaneously was 98.82%. These results indicated that administration via subcutaneous injection prolonged the extension time of ZGS1 in vivo when compared to intravenous administration and increased the AUC_0–t_ of the active metabolite ZGS2. The calculation formula of AUC, MRT and t_1/2_ is as follows:

AUC(0−t)=∫0t*Cdt+∫t*∞Cdt t12z=0.693/K MRT(0−t)=∫0∞tcdt∫0∞cdt=AUMCAUC

### 2.5. Tissue Distribution 

**Figure 6:**
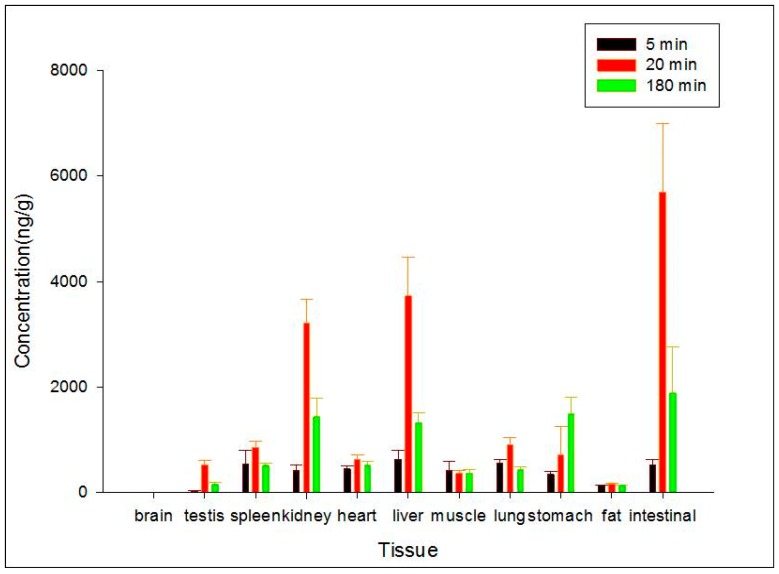
Concentration (mean ± SD) levels of ZGS1 in rat tissue at 5, 20, and 180 min after subcutaneous injection injection of ZGS1 (10 mg/kg) (*n* = 5).

**Figure 7:**
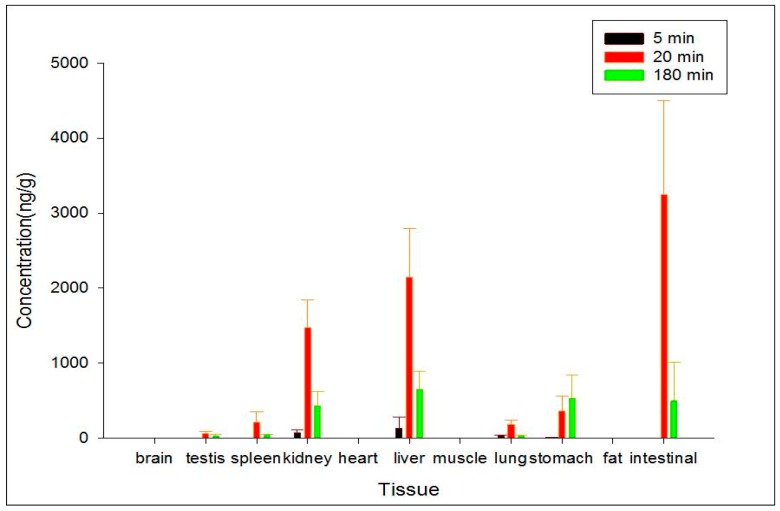
Concentration (mean ± SD) levels of ZGS2 in rat tissue at 5, 20, and 180 min after subcutaneous injection of ZGS1 (10 mg/kg) (*n* = 5).

Administration by subcutaneous injection was chosen to evaluate the distributions of ZGS1 and ZGS2. The distribution of ZGS1 and ZGS2 in different tissues at 5, 20, and 180 min after subcutaneous administration of ZGS1 were assessed (Figure 6 and Figure 7). The standard curves of ZGS1 and ZGS2 in different tissues are shown in Table 2 and Table 3. ZGS1 was rapidly and widely distributed in most organs, except for the brain. 

Levels of ZGS1 and ZGS2 were lower in brain tissue, when compared to other tissues, suggesting that ZGS1 and ZGS2 do not easily cross the blood brain barrier (BBB). In addition, the highest levels of ZGS1 and ZGS2 were observed in the intestine, followed by the liver, and kidney at 20 min.

### 2.6. Excretion Study

**Figure 8:**
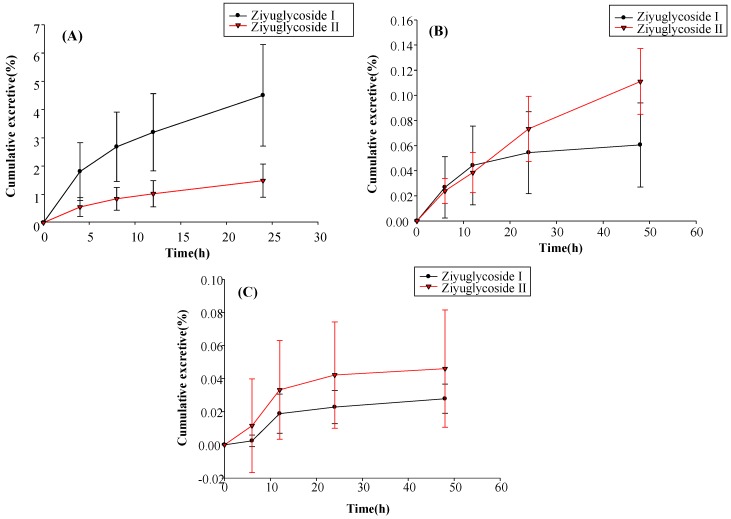
(**A**) Biliary; (**B**) urinary; and (**C**) fecal cumulative excretion profiles of ZGS1 and ZGS2 in rats after subcutaneous injection of ZGS1 (10 mg/kg) (*n* = 5).

The excretion of ZGS1 and ZGS2 in rat urine, feces, and bile was determined after subcutaneous administration of ZGS1 (10 mg/kg) (Figure 8). The mean recoveries of unchanged ZGS1 were 5.990%, 0.172%, and 0.074% of the 10 mg/kg dose up to 24 h in bile, urine, and fecal samples, respectively.

These results indicated that ZGS1 was primarily excreted by the bile, and supported the results that ZGS1 was mostly present in rat intestines. However, further studies are required to investigate the in vivo biotransformation of ZGS1.

## 3. Materials and Methods 

### 3.1. Chemical, Reagents, and Materials

ZGS2 and α-hederin (internal standard (IS), HPLC > 98%) were extracted and purified in-house. ZGS1 (HPLC > 98%) was purchased from Raffensi Biotechnology (Chengdu, China). Methanol and acetonitrile (HPLC-MS grade) were purchased from Thermo Fischer Scientific (Fair Lawn, NJ, USA). Formic acid (mass spectrometer grade) was purchased from Aladdin Corporation (Shanghai, China), and Milli-Q ultrapure water was obtained from a Millipore system (Milford, MA, USA).

### 3.2. LC-MS Conditions

The liquid chromatography (LC) system used was comprised of a binary pump, a constant temperature column chamber, an auto sampler, and a diode array detector (DAD). An XTERRA MS C_18_ reversed phase column (2.1 × 50 mm, 5 µm) was used for the determination of analytics. The temperature of the column was maintained at 40 °C. The mobile phase A (MA) was water, containing 0.2% formic acid (FA) (*v*/*v*) and mobile phase B (MB) contained acetonitrile. The flow velocity was set to 0.4 mL/min. In 6 min, the gradient elution was set to: 0.0–0.1 min, 10% MB; 0.1–1 min, 10% MB; 1–1.5 min, 10–57% MB; 1.5–2.0 min, 57–68% MB; 2.0–3.2 min, 68–75% MB; 3.2–4.0 min, 75–95% MB; 4.0–4.5 min, 95–10% MB; 4.5–6 min, 10% MB. The injection volume was 2 µL. For better stability of the samples, the auto-sampler was kept at a temperature of 4 °C.

An Agilent 1290 Infinity Rapid Resolution Liquid Chromatography (RRLC) System (Agilent, Lexington, MA, USA), coupled to an Agilent 6460 triple quadrupole mass spectrometer was used for quantitative measurements. Electrospray ionization (ESI) negative and multiple reaction monitoring (MRM) ion mode was used for detection of the ions. The optimal conditions for mass spectrometry were as follows: capillary voltage (CV) 3000 V, gas flow rate 5 L/min, dry gas temperature 300 °C, sheath gas temperature 250 °C, dry flow rate 7 L/min, atomizer pressure 45 psi, and instrument use process using nitrogen. For ZGS2, the precursor-product ion pair was *m*/*z* 811.4→602.9, for ZGS1, *m*/*z* 603.4→585.2, and for α-hederin (IS) *m*/*z* 749.3→471.1 ZGS1, ZGS2, and IS fragments were obtained at −237 V, −330 V, and −320V, respectively. The collision energy (CE) of ZGS1, ZGS2, and IS was −27 eV, −35 eV, and −50 eV, respectively. MassHunter (version B.04) software was used for analysis and data acquisition. Multiple-reaction monitoring was used for further detection. The detailed characteristics are shown in Table 1.

### 3.3. Sample Preparation

For preparation of the stock solution (SS), calibration standards, and quality control (QC) samples, ZGS1, ZGS2 as well as α-hederin were dissolved in methanol to a final concentration of 1 mg/mL. Working solutions of calibration standards and QC samples of ZGS1 and ZGS2 were prepared by dilution with methanol (containing 0.1% FA). Similarly, the α-hederin IS was further diluted with methanol (containing 0.1% FA) to a final concentration of 800 ng/mL. For ZGS1 and ZGS2, two stock solutions were prepared, one for the calibration standards, the other for QC samples. Stock solutions were stored at 4 °C.

To achieve a calibration concentration range from 5 to 2000 ng/mL, including 5, 20, 50, 100, 500, 1000, and 2000 ng/mL, stock and working solutions were added to biological samples. For ZGS1 and ZGS2, QC samples of 10, 800, and 1600 ng/mL were prepared. Working solutions for calibration standards of ZGS1 and ZGS2 and QC samples of ZGS1 and ZGS2 were stored at 4 °C for future use.

### 3.4. Preparation of Biological Samples

A total of 20 μL IS working solution was added to 50 μL plasma, urine, and bile samples. Then, 330 µL methanol (containing 0.1% FA) was added to precipitate the proteins in the samples. After vortex-mixing for 5 min, samples were centrifuged at 12,000 rpm for 5 min at 4 °C. Then, HPLC-MS/MS analysis was used to analyze the supernatant (200 µL). Tissue samples were homogenized in water at a ratio of 1:4 (*w*/*v*), while pulverized 0.2 g of fecal material was homogenized with water (1:5, *w*/*v*). A total of 50 μL suspension was analyzed.

### 3.5. Method Validation

The methodology used in this study was performed as per FDA guidelines [19]. The HPLC-MS/MS analysis for ZGS1 and ZGS2 was validated at a range of 5–2000 ng/mL. Parameters of selectivity, including a lower limit of quantification (LLOQ), matrix effects (ME), precision, accuracy, recovery, stability, and dilution reliability of this method were fully validated. 

The specificity of endogenous substances and analytes in rat plasma was determined by determining the chromatograms of plasma samples in six rats. The standard plasma samples used for creating of the calibration curves were assayed. By plotting the ratio of analyte/IS peak area versus the ratio of the nominal concentration, calibration curves were constructed using linear regression (y = ax + b, weighting factor of 1/*x*^2^). Acceptance criteria for the LLOQ were ±20% with a correlation coefficient (r^2^) greater than 0.99. In addition, the acceptable limit of the relative standard deviation (RSD) for each point in the standard curve was ±15%.

Precision and accuracy (Intra-day) assay were evaluated by analyzing replicates (*n* = 6) at QC levels of 10 (Low quality control, LQC), 800 (Middle quality control, MQC) and 1600 (High quality control, HQC) ng/mL, respectively. Moreover, precision and accuracy (Inter-day) from QCs samples were evaluated at 3 consecutive days. Sample precision and accuracy were assessed by RSD and relative error (RE). The maximum acceptable deviation was ±15%.

To determine the extraction recovery, the peak area ratio extracted from 10 ng/mL (LQC), 800 ng/mL (MQC), and 1600 ng/mL (HQC) QC samples as well as the peak areas that ZGS1 and ZGS2 spiked to the blank sample extracts were calculated at the same concentrations. The ME were determined by matrix factor (MF), which presented the ratio of the ZGS1 and ZGS2 peak response in the presence of matrix ions to the analyte peak response in methanol. Experiments were performed in six replicates at 10 ng/mL, 800 ng/mL and 1600 ng/mL levels.

The stability of ZGS1 and ZGS2 in rat plasma was assessed by analyzing replicates (*n* = 5) at LQC, MQC, and HQC levels. The short-term stability of the standards and QC samples of ZGS1 and ZGS2 was assessed by evaluating the samples after 24 h at room temperature. The freeze–thaw stability was evaluated by subjecting LQC, MQC, and HQC samples to three freeze–thaw cycles prior to extraction. Samples were stored at −80 °C for 30 days. Then, samples were thawed to room temperature, and analyzed by the approach described above. The analytes were considered stable in plasma when the concentrations were within 85–115% of the initial concentration.

To obtain ZGS1 and ZGS2 samples within the calibration range of 5–2000 ng/mL, samples were diluted using the highest concentration (20,000 ng/L 10-times, the upper limit of quantification (ULOQ)) of the QC sample, which was diluted with plasma. 

### 3.6. Pharmacokinetic Data Analysis

Ten male Sprague-Dawley (SD) rats weighing 150–200 g were purchased from the Hunan Shrek Laboratory Animal (Changsha, China). All experimental procedures involving animals were reviewed and approved by the Animal Care and Use Committee (ACUC) of Jiangxi University of Traditional Chinese Medicine (Nanchang, China) and were in accordance with the Guide for the Care and Use of Laboratory Animals (GCULA). Previous pharmacodynamic studies have shown that in rats, administration of 10 mg/kg ZGS1 has the best beneficial effects. Therefore, we chose to use this dose for our future studies. The general health status of rats was observed daily. Rats were fasted for 12 h but had with free access to water prior to experiments. Rats were randomly divided into two groups, one group received tail vein injection 10 mg/kg ZGS1, the other group received a subcutaneous injection of 10 mg/kg ZGS1. Approximately 0.15 mL of rat blood samples were collected from the orbital venous plexus at the following time points: pre-dose, 0.083, 0.333, 0.666, 1, 1.333, 1.667, 2, 3, 4, and 6 h. Plasma was prepared by centrifugation at 4000× *g* for 5 min at 4 °C. The supernatant was separated and stored at −40 °C until sample preparation and analysis. Non-compartmental analysis of the data was acquired and processed by using DAS 3.0 software (Chinese Pharmacological Society). Results are expressed as the mean ± standard deviation (SD).

### 3.7. Tissue Distribution Study

To study the target distribution of ZGS1 and ZGS2, tissue distribution was evaluated. In brief, 15 rats (150–200 g) were randomly divided into three groups. After subcutaneous injection of 10 mg/kg of ZGS1, 11 tissues, including liver, heart, spleen, lung, brain, intestine, stomach, kidney, testis, skeletal muscle, and fat were collected at 5, 20, or 180 min. Tissues were immediately rinsed with normal saline solution, dried, and accurately weighed. Samples were homogenized following the methods described above and stored at −40 °C until LC-MS analysis.

### 3.8. Excretion Study

For the excretion study, 12 rats were randomly divided into two groups. One group was used for the urinary and fecal excretion study, whereas the other group was used for evaluating bile excretion. All animals had free access to food and water. Blank samples for urine, feces, and bile were collected before animals were treated. After subcutaneous injection of 10 mg/kg of ZGS1, rats were housed in metabolic cages, and urine and fecal samples were collected at 0–6 h, 6–12 h, 12–24 h, and 24–48 h. Fecal samples were dried at room temperature. After the dry weight of fecal samples and the volume of urine for each collection point were measured, specimens were stored at −40 °C. Rats in the group for bile excretion evaluation were anesthetized with 20% urethane and a cannula was implanted into the bile duct to collect bile. Bile samples were collected at 0–4 h, 4–8 h, 8–12 h, and 12–24 h, and stored at −40 °C.

Investigating the QC samples in the tissue homogenates was sufficient and equivalent to the peak area measured by the plasma samples under the same condition. Therefore, the above findings indicated that the method of plasma samples can be used to determine the content of ZGS1 and ZGS2 in other tissues.

## 4. Conclusions

In this study, a simple and sensitive HPLC–MS/MS method was developed for the quantification of ZGS1 and ZGS2 in rat biological matrices. The PK, tissue distribution, and excretion of ZGS1 and ZGS2 were evaluated. In this study, we demonstrated that ZGS1 and ZGS2 can rapidly be cleared from rat plasma following tail vein injection and subcutaneous injection of ZGS1. The major organs of ZGS1 and ZGS2 distribution included the intestines, liver, and kidney. The total recovery of ZGS1 in bile, urine, and feces were low, and may potentially be due to in vivo biotransformation. In vivo processing of the ZGS2 metabolite may form the basis of the effect of ZGS1 on leucopenia.

According to the novel drug Research and Development requirement of the FDA and the CFDA (China), the PK profile of the prototype and the metabolite is acritical measure in the search for drug candidates. Several studies have focused on PK profile of prototypes and metabolites [20,21,22,23]. Previous studies have suggested that, ZGS2 is the main metabolite of ZGS1, and to better understand its beneficial effects in vivo may help to increase the success rate of potential drugs. In our previous study, we indicated that ZGS1 and ZGS2 possessed remarkable activity on myelosuppression mice. To our knowledge, this is the first time that the PK profile of ZGS1 and its metabolite ZGS2 were studied. Therefore, ZGS1 is a potential candidate for the development of novel drugs to treat leucopenia.

## Figures and Tables

**Table 1:** Retention time (t_R_), optimized MS/MS transitions, fragmentor voltage (FV) and collision energy (CE) for each analyte.

Analytes	t_R_ (min)	Precursorion	Production	FV (eV)	CE (eV)
Ziyuglycoside I	2.08	811.4	602.9	237	27
Ziyuglycoside II	2.39	603.5	585.3	330	35
α-hederin (IS)	2.32	749.3	471.1	320	50

**Table 2:** Linearity and Linear range of ZGS1 calibration curves in different biological matrix.

Matrix	Calibration Curve	Correlation Coefficient (r^2^)	Linear Range (ng/mL)
plasma	y = 0.010x − 0.031	0.999	5–2000
brain	y = 0.001x − 0.003	0.999	5–2000
testis	y = 0.001x + 0.001	0.999	5–2000
spleen	y = 0.001x − 0.009	0.998	5–2000
kidney	y = 0.001x + 0.001	0.999	5–2000
heart	y = 0.001x − 0.007	0.999	5–2000
intestinal	y = 0.001x − 0.006	0.998	5–2000
muscle	y = 0.001x − 0.003	0.999	5–2000
liver	y = 0.001x − 0.003	0.999	5–2000
lung	y = 0.001x − 0.007	0.999	5–2000
stomach	y = 0.001x − 0.005	0.999	5–2000
fat	y = 0.001x − 0.012	0.999	5–2000
bile	y = 0.001x − 0.005	0.998	5–2000
Urine	y = 0.001x − 0.011	0.999	5–2000
feces	y = 0.002x + 0.010	0.999	5–2000

**Table 3:** Linearity and Linear range of ZGS2 calibration curves in different biological matrix.

Matrix	Calibration Curve	Correlation Coefficient (r^2^)	Linear Range (ng/mL)
plasma	y = 0.010x + 0.569	0.996	5–2000
brain	y = 0.001x + 0.057	0.993	5–2000
testis	y = 0.001x + 0.040	0.996	5–2000
spleen	y = 0.001x + 0.043	0.996	5–2000
kidney	y = 0.001x + 0.054	0.992	5–2000
heart	y = 0.001x + 0.065	0.994	5–2000
intestinal	y = 0.001x + 0.039	0.995	5–2000
muscle	y = 0.001x + 0.052	0.992	5–2000
liver	y = 0.001x + 0.059	0.991	5–2000
lung	y = 0.001x + 0.041	0.996	5–2000
stomach	y = 0.001x + 0.042	0.996	5–2000
fat	y = 0.001x + 0.039	0.997	5–2000
bile	y = 0.001x + 0.017	0.998	5–2000
Urine	y = 0.001x − 0.007	0.999	5–2000
feces	y = 0.001x + 0.011	0.999	5–2000

**Table 4:** Intra- and inter-day accuracy and precision of ZGS1 and ZGS2 in rat plasma (*n* = 5).

Compound	Concentration (ng/mL)	Intra-Day (*n* = 5)		Inter-Day (*n* = 5)	
		Accuracy (RE, %)	Precision (RSD, %)	Accuracy (RE, %)	Precision (RSD, %)
Ziyuglycoside I	10	4.81	2.42	6.00	4.12
	800	1.89	1.14	2.41	2.65
	1600	1.23	3.31	1.00	4.48
Ziyuglycoside II	10	1.28	4.36	1.76	3.99
	800	1.75	0.81	2.77	0.95
	1600	1.18	1.94	3.58	1.02

**Table 5:** Extraction recovery and matrix effect of ZGS1 and ZGS2 in rat plasma (*n* = 5).

Compound	Concentration (ng/mL)	Recovery (%)	RSD (%)	IS Normalized Matrix Factor (%)	RSD (%)
Ziyuglycoside I	10	96	3.63	105	5.26
	800	96	2.76	104	3.22
	1600	98	2.42	103	4.27
Ziyuglycoside II	10	102	4.09	100	7.55
	800	98	4.60	106	4.24
	1600	100	3.68	107	2.27
α-hederin (IS)	800	94	2.71		

**Table 6:** Stability data of ZGS1 and ZGS2 in rat plasma (*n* = 5).

Compound	Concentration	Short-Term Stability for 24 h (25 °C)		3 Freeze-Thaw Cycles Stability		Long-Term Stability for 2 Weeks	
	(ng/mL)	RE (%)	RSD (%)	RE (%)	RSD (%)	RE (%)	RSD (%)
Ziyuglycoside I	10	3.48	5.19	5.65	9.65	5.07	9.28
	800	2.21	1.59	3.79	1.93	2.87	3.54
	1600	2.18	1.09	2.53	1.45	2.13	2.79
Ziyuglycoside II	10	4.22	4.86	5.95	12.77	6.84	9.61
	800	3.86	2.66	4.21	3.83	3.86	5.07
	1600	2.37	3.07	2.65	5.72	1.81	5.80

**Table 7:** Pharmacokinetic parameters of ZGS1 and ZGS2 in rats after tail vein administration of 10 mg/kg ZGS1 (*n* = 5).

Parameter	Unit	Ziyuglycoside I (Mean ± SD)	Ziyuglycoside II (Mean ± SD)
AUC_(0–t)_	μg/L * h	534.841 ± 73.569	299.6 ± 28.083
MRT_(0-t)_	h	0.421 ± 0.061	0.575 ± 0.06
t_1/2_z	h	1.338 ± 0.744	1.027 ± 0.057

**Table 8:** Pharmacokinetic parameters of ZGS1 and ZGS2 in rats after subcutaneous injection administration of 10 mg/kg ZGS1 (*n* = 5).

Parameter	Unit	Ziyuglycoside I (Mean ± SD)	Ziyuglycoside II (Mean ± SD)
AUC_(0-t)_	μg/L * h	528.558 ± 91.819	421.161 ± 74.152
MRT_(0-t)_	h	2.196 ± 0.173	2.436 ± 0.263
t_1/2_z	h	6.115 ± 1.92	7.935 ± 3.264
Tmax	h	0.333	0.333
Vz/F	L/kg	94.437 ± 25.52	117.41 ± 28.764
CLz/F	L/h/kg	10.999 ± 1.839	10.996 ± 2.802
Cmax	ng/mL	303.257 ± 93.74	177.652 ± 51.225

## References

[1] Lyman G.H. (2016). Issues on the Use of White Blood Cell Growth Factors in Oncology Practice. Am. Soc. Clin. Oncol. Educ. Book.

[2] Fagnani D., Isa L., Verga M.F., Nova P., Casartelli C., Filipazzi V., Danova M., Farina G., Pugliese P., Fava S. (2014). Granulocyte colony-stimulating factors used in clinical practice: PoloNord Registry-Based Cohort Italian Study. Tumori.

[3] Khoury H.A., Adkins D., Brown R., Vij R., Westervelt P., Teinkaus K., Goodnough L.T., Dipersio J.F. (2000). Adverse side-effects associated with G-CSF in patients with chronic myeloid leukemia undergoing allogeneic peripheral blood stem cell transplantation. Bone Marrow Transplant..

[4] Natarajan S., Narayan S., Yin J.A.L., Chan A. (2012). The Use of Prophylactic Granulocyte-colony Stimulating Factor for Chemotherapy-induced Febrile Neutropenia. Eur. Oncol. Haematol..

[5] Weng Y.J., Jiang D.X., Liang C.X., Gastroenterology D.O. (2015). Research of Diyushengbai tablets in prevention of immune function decline after FOLFOX chemotherapy for patients with colorectal carcinoma. China Pract. Med..

[6] Li H., Yang F. (2007). Observation on Diyushengbai tablet to prevent myelosupression of patients treated by chemotherapy. China Med. Herald.

[7] Liu C.Y., Hu X.Y., Xu L.X., Kong Y., Liu F.L. (2015). Clinical analysis of Diyu Shengbai tablets combined with granulocyte colony stimulating factor in treatment of cancer related fatigue syndrome. Chin. J. Biol. Pharm..

[8] Cai Z., Li W., Wang H., Yan W., Zhou Y., Wang G., Cui J., Wang F. (2012). Anti-tumor and immunomodulating activities of a polysaccharide from the root of *Sanguisorba officinalis* L.. Int. J. Biol. Macromol..

[9] Zhang L., Koyyalamudi S.R., Jeong S.C., Reddy N., Smith P.T., Ananthan R., Longvah T. (2012). Antioxidant and immunomodulatory activities of polysaccharides from the roots of *Sanguisorba officinalis*. Int. J. Biol. Macromol..

[10] Ming B., Zhang S. (2015). Clinical Study on Diyu Shengbai Tablets to Prevent Peripheral Blood Leukcyte Reduction Caused by Chemoradiotherapy in Patients with Nasopharyngeal Carcinoma. J. Liaoning Univ. Tradit. Chin. Med..

[11] Zhang R., Tang Y., Yu L., Wu B. (2012). A systematic review of Garden burnet root leukopoietic tablets treatment and prevention of radiotheraphy-induced leucopenia. Chin. Hosp. Pharm..

[12] Chen X., Li B., Gao Y., Ji J.X., Wu Z.L., Chen S. (2017). Saponins from *Sanguisorba officinalis* Improve Hematopoiesis by Promoting Survival through FAK and Erk1/2 Activation and Modulating Cytokine Production in Bone Marrow. Front. Pharmacol..

[13] Wang G.J., Fu H., Ye W., Zheng X., Xiao J., Kang D., Rao T., Shao Y., Xie L., Liang Y. (2016). Comprehensive characterization of the in vitro and in vivo metabolites of ziyuglycoside I in rat microsome, intestinal flora, excretion specimen and fresh tissues based on LC-Q-TOF/MS. J. Pharm. Biomed. Anal..

[14] Zhu A.K., Zhou H., Xia J.Z., Jin H.C., Wang K., Yan J., Zuo J.B., Zhu X., Shan T. (2013). Ziyuglycoside II-induced apoptosis in human gastric carcinoma BGC-823 cells by regulating Bax/Bcl-2 expression and activating caspase-3 pathway. Braz. J. Med. Biol. Res..

[15] Nam S.H., Lkhagvasuren K., Seo H.W., Kim J.K. (2017). Antiangiogenic Effects of Ziyuglycoside II, a Major Active Compound of *Sanguisorba officinalis* L.. Phytother. Res..

[16] Xiao J.C., Chen H.M., Fu H.X., Ye W., Rao T., Shao Y.H., Kang D., Shen B.Y., Xie L., Wang G.J. (2016). Development of a novel sectional multiple filtering scheme for rapid screening and classifying metabolites of ziyuglycoside II in rat liver and excreta specimen based on high-resolution mass spectrometry. J. Pharm. Biomed. Anal..

[17] Ye W., Fu H.X., Xie L., Zhou L.J., Rao T., Wang Q., Shao Y.H., Xiao J.C., Kang D., Wang G.J. (2015). Development and validation of a quantification method for ziyuglycoside I and II in rat plasma: Application to their pharmacokinetic studies. J. Sep. Sci..

[18] Kadian N., Raju K.S.R., Rashid M., Mamunur R., Malik M.Y., Taneja I. (2016). Comparative assessment of bioanalytical method validation guidelines for pharmaceutical industry. J. Pharm. Biomed. Anal..

[19] Agency E.M. (2012). Guideline on bioanalytical method validation. Drug Eval. Res..

[20] Chen Y., Guo J., Tang Y., Wu L., Tao W., Qian Y., Duan J.A. (2015). Pharmacokinetic profile and metabolite identification of yuanhuapine, a bioactive component in *Daphne genkwa* by ultra-high performance liquid chromatography coupled with tandem mass spectrometry. J. Pharm. Biomed. Anal..

[21] Alamri R.G., Mohsin K., Ahmad A., Raish M., Alanazi F.K. (2017). Development and validation of bioanalytical UHPLC-UV method for simultaneous analysis of unchanged fenofibrate and its metabolite fenofibric acid in rat plasma: Application to pharmacokinetics. Saudi Pharm. J..

[22] Xu X.W., Su X.J., Zhang Y.N., Zheng X.K., Lv P.F., Hu J. (2015). Simultaneous determination of nintedanib and its metabolite BIBF 1202 in different tissues of mice by UPLC–MS/MS and its application in drug tissue distribution study. J. Chromatogr. B.

[23] Lin D., Qiao L.M., Zhang Y.N., Liu Y., Liu X.S. (2016). Simultaneous determination of nintedanib and its metabolite byUPLC–MS/MSin rat plasma and its application to a pharmacokineticstudy. J. Pharm. Biomed. Anal..

